# The Prevalence and Incidence of Suicidal Thoughts and Behavior in a Smartphone-Delivered Treatment Trial for Body Dysmorphic Disorder: Cohort Study

**DOI:** 10.2196/63605

**Published:** 2025-05-06

**Authors:** Adam C Jaroszewski, Natasha Bailen, Simay I Ipek, Jennifer L Greenberg, Susanne S Hoeppner, Hilary Weingarden, Ivar Snorrason, Sabine Wilhelm

**Affiliations:** 1 Department of Psychiarty, Massachusetts General Hospital, Harvard Medical School, Simches Research Building, 185 Cambridge Street, Boston, MA, 02114, United States, 1 617-724-6300; 2 Department of Psychology, Ferkauf Graduate School of Psychology, Yeshiva University, New York, NY, United States

**Keywords:** suicide, body dysmorphic disorder (BDD), smartphone-delivered, digital mental health intervention (DMHI), adult, suicidal thoughts and behavior, suicidal, self-harm, self-injurious, smartphone, treatment, medication, suicide prevention, digital mental health, digital health, mental health, prevalence, risk assessment, burden, mobile health, mHealth, health informatics

## Abstract

**Background:**

People with past suicidal thoughts and behavior (STB) are often excluded from digital mental health intervention (DMHI) treatment trials. This may perpetuate barriers to care and reduce treatment generalizability, especially in populations with elevated rates of STB, such as body dysmorphic disorder (BDD). We conducted a cohort study of randomized controlled trial (RCT) participants (N=80) who received a smartphone-based cognitive behavioral therapy (CBT) treatment for BDD that allowed for most forms of past STB, except for past-month active suicidal ideation.

**Objective:**

This study had two objectives: (1) to characterize the sample’s lifetime prevalence of STB and (2) to estimate and predict STB incidence during the trial.

**Methods:**

We completed secondary analyses on data from an RCT of smartphone-delivered CBT for BDD. The primary outcomes consisted of STB severity and suicide attempt assessed at baseline with the Columbia-Suicide Severity Rating Scale (C-SSRS) and weekly during the trial via one item from the Quick Inventory of Depressive Symptomatology-Self Report (QIDS-SR item #12; 1043 observations). We computed descriptive statistics (n, %) and ran a series of bi- and multivariate linear regressions predicting STB incidence during the 3-month trial.

**Results:**

At baseline, 40% of participants reported a lifetime history of active suicidal thoughts and 10% reported lifetime suicide attempts. During the 3-month trial, 42.5% reporting thinking about death or suicide via weekly assessment. No participants reported frequent or acute suicidal thoughts, plans, or attempts. Lifetime suicide attempt (odds ratio 11, 95% CI 2.14‐59.14; *P*<.01) and lifetime severity of suicidal thoughts (odds ratio 1.76, 95% CI 1.21‐2.77; *P*<.01) were significant bivariate predictors of death- or suicide-related thought incidence reported during the trial. Multivariate models including STB risk factor covariates (eg, age, and sexual orientation) modestly improved prediction of death- or suicide-related thoughts (eg, positive predictive value=0.91, negative predictive value=0.75, and area under the receiver operating characteristic curve=0.83).

**Conclusions:**

Although some participants may think about death and suicide during a DMHI trial, it may be safe and feasible to include participants with most forms of past STB. Among other procedures, researchers should carefully select eligibility criteria, use frequent, ongoing, low-burden, and valid monitoring procedures, and implement risk mitigation protocols tailored to the presenting problem.

## Introduction

Mental illness directly affects nearly half of all adults in the United States [1]. Although effective treatments have been developed for a wide range of disorders, demand far outstrips availability [2]. Indeed, care gaps are estimated at above 50% [3]. Some barriers to accessing mental health care include the limited number of clinicians, high cost of services, limited insurance coverage, and entrenched stigma. Digital mental health interventions (DMHIs) promise to address many of these concerns. DMHIs can be relatively inexpensive, reduce clinician time, and may reduce stigma through increased privacy [4,5]. Participants in DMHI trials often compete study components remotely, asynchronously, and relatively independently [6,7]. The light-touch nature of many DMHIs presents issues for researchers designing treatment trials, who might wonder if they are safe for people with higher clinical acuity, such as those with past or current suicidal thoughts and behavior (STB), as past STBs are the strongest known risk factors for future suicide thoughts, attempt, and death [8,9]

People with past and current STB are often excluded from treatment studies, including those testing DMHIs [6,10,11]. Indeed, recent meta-analytic reviews of depression and anxiety DMHI trials have found that between 60% to 90% of studies contained exclusion criteria based on “suicide risk” [6,7] with 33% of computerized depression and anxiety trials excluding people with “current” suicidal ideation, plan, or intent, 70% excluding based on recent-past suicidal thoughts (eg, thoughts occurring in the past 2 weeks, but not necessarily at the time of index assessment), and nearly 15% excluding based on a reported “history of suicidality” (eg, suicide attempt) [6]. Among the handful (n=5) of body dysmorphic disorder (BDD)–focused DMHI trials (excluding the presently discussed trial [12]), most used a past-STB–related exclusion criterion, too—specifically, either past-week or month active or acute suicidal ideation (n=2; [13, 14]) or past suicide attempt (n=2; [15, 16]). Because it is difficult to accurately predict STB [8,17] and few studies report on STB incidence during a trial or provide details on the content of adverse events [18], it is difficult for researchers to establish appropriate evidence-based STB exclusion criteria versus likely overly restrictive criteria.

Trials with appropriately restrictive STB exclusion criteria may mitigate (but likely not eliminate) risks associated with adverse events and reduce time and effort associated with managing unduly high STB incidence while also allowing for a relatively representative sample. By contrast, trials with overly restrictive STB criteria may more effectively reduce these risks but, crucially, would also likely reduce real-world generalizability and reproducibility. Also, overly restrictive STB exclusionary practices may perpetuate endemic barriers to care that people with STB already experience in face-to-face clinical research studies and real-world treatment settings [19].

The purpose of the present study was to describe the suicide risk mitigation procedures used in a recent randomized controlled trial (RCT) of smartphone-delivered cognitive behavioral therapy (CBT) for BDD and to describe and predict the actual occurrence of STBs during the 12-week trial. This study provides a relatively strong test of the risk mitigation procedures followed because (1) in general, people with BDD have high rates of suicidal thoughts [20,21,22,23], suicide attempts [eg, 21, 24, 25], and completed suicides [21]; (2) although the current RCT excluded based on “active” suicidal thoughts occurring in the past month, it did not exclude any form of STB occurring>30 days before baseline assessment, including past suicide attempt, active suicidal ideation, plans, and intent, which makes it less restrictive than other BDD focused DMHI trials; and (3) participants with some forms of recent (ie, ≤30 d before baseline) death- or suicide-related thoughts were still eligible to participate (eg, wish to be dead).

## Methods

### Overview

A randomized waitlist-controlled trial was conducted with 80 adults with a primary BDD diagnosis. The treatment group had access to the Perspectives BDD, a coach-guided smartphone app that provided CBT for BDD for 12 weeks [12]. Perspectives BDD included modules on psychoeducation, restructuring maladaptive thoughts, exposure with response prevention, mindfulness and attentional retraining, values and enhancing self-esteem and self-compassion, and relapse prevention. Participants also had access to bachelor’s-level coaches who aimed to enhance motivation and engagement via two telephone calls (onboarding and midtreatment) along with asynchronous in-app text-based messaging throughout the treatment period. Participants answered brief (four-item) weekly in-app surveys related to their current symptom levels and suicidal thoughts. They were also assessed by a trained doctoral-level clinician at screening and baseline, mid (week 6), and end-of-treatment (week 12). Participants initially allocated to the waitlist received access to the app-based treatment after 3 months. Weekly data collected from participants while on the waitlist is included and analyzed along with all data collected during active treatment period.

To be eligible for the study, individuals were required to have a primary BDD diagnosis, be at least 18 years old, and reside in the United States. Individuals were excluded if they were currently in therapy, had previously completed 4 or more sessions of CBT for BDD, had undergone psychotropic medication changes less than 2 months before starting the study, reported acute and active suicidal ideation (refer to “Procedures Used for Mitigating Clinical Deterioration and Suicide Risk” section below), or were unable to engage with the smartphone treatment. Other exclusion criteria included a current severe substance use disorder, current severe comorbid major depressive disorder, or a lifetime bipolar or psychotic disorder. The sample was predominantly female (84%), non-Hispanic (88%), and White (71%) with a mean age of 27 (SD 9.6; refer to ClinicalTrials.gov NCT04034693 trial registration and [12] for additional information regarding the treatment, participants, and study design and procedures). To reduce burden, demographic information was not collected from people excluded from participating in the trial. All procedures were approved by the Mass General Brigham Institutional Review Board.

### Measures

#### Clinician-Administered

Relevant clinician-administered measures included the Yale-Brown Obsessive Compulsive Scale Modified for BDD (BDD-YBOCS; [26]), Mini International Neuropsychiatric Interview (MINI 7.02; [27]), and the Columbia-Suicide Severity Rating Scale (C-SSRS [28]; refer to [12] for additional measures administered). The BDD-YBOCS is the gold-standard measure of BDD symptom severity and was used to characterize the sample and track symptom severity. The MINI, a standardized diagnostic interview, was used to determine *DSM-5* (*Diagnostic and Statistical Manual of Mental Disorders* [Fifth Edition]) psychiatric diagnoses and to evaluate whether BDD was primary. The C-SSRS was used to assess lifetime and past 30-day suicidal ideation, intent, and behavior. Measures were administered at baseline by blinded, doctoral-level independent evaluators.

#### Self-Report

Relevant self-report measures include the Quick Inventory of Depressive Symptomatology-Self Report (QIDS-SR; [29]) and the Clinical Global Impressions scale [30] adapted for assessing BDD symptoms (CGI-BDD), and the Patient Health Questionnaire-2 (PHQ-2; [31]).

The QIDS-SR and CGI-BDD were administered at baseline, mid, and end-of-treatment assessments. To assess past-week death- and suicide-related thought severity, item #12 of the QIDS-SR was administered to participants via weekly in-app surveys and via web-based surveys to those on the waitlist. The item’s label was: “Thoughts of Death and Suicide.” Answer choices included: 0 (“I do not think of suicide or death”), 1 (“I feel that life is empty or wonder if it’s worth living”), 2 (“I think of suicide or death several times a week for several minutes”), and 3 (“I think of suicide or death several times a day in some detail, or I have made specific plans for suicide or have actually tried to take my life”).

An item from the CGI-BDD was used to determine whether participants perceived their past-week BDD symptoms improving or worsening. Participants rated their current symptoms “compared to how [they] felt when first entering the study.” Answer choices ranged from 1 (very much improved) to 7 (very much worse). The PHQ-2 was used to assess core diagnostic symptoms of depression severity (low mood and anhedonia), answer choices ranged from 0 (not at all) to 3 (every day). The QIDS-item #12, CGI-BDD and PHQ-2 were used as indicators of suicide and psychiatric risk, which triggered the risk-mitigation procedures detailed in “During the Trial” section.

### Procedures Used for Mitigating Clinical Deterioration and Suicide Risk

#### During Screening

Exclusion criteria sought to limit participants with “very severe depression” (≥21 on the QIDS-SR; [27]) and active, acute suicidal ideation (≥2 on the C-SSRS suicide ideation severity subscale for past-month suicidal thoughts). Prospective participants endorsing these symptoms were contacted by a doctoral-level independent evaluator, who followed up with the participant via phone within 24 hours to evaluate safety and make a referral to a higher level of care if clinically indicated.

#### During the Trial

The app homepage presented a reminder to participants that if they were experiencing suicidal thoughts, they should go to the emergency room right away. Links to 911 and the national suicide hotline were also presented. Participants designated an emergency contact (eg, friend or relative) in case that the participant was unreachable, and the study team was concerned about their well-being. Also, participants could be withdrawn if, in the judgment of the principal investigator (PI), remaining in the study posed a substantial risk to the participant or a higher level of psychiatric care was needed due to significant clinical deterioration or active, acute suicidal thoughts.

Participants’ clinical deterioration and suicide thoughts or behaviors were assessed weekly via a brief in-app survey and at study mid and endpoints via self-report. Clinical deterioration was defined by a combination of (1) a rating of 6 (much worse) or 7 (very much worse) on the weekly, participant-rated CGI-BDD across 2 subsequent weeks and (2) PI judgment that remaining in the study was not clinically indicated. Of note, a single rating of 6 or 7 on the weekly, participant-rated CGI-BDD triggered an alert to the clinician and coach via SMS text message or email, which was followed up by a licensed, doctoral-level study clinician within 24 hours of the alert. Study clinicians received supervision by the PI and Coinvestigators, all of whom had decades of experience conducing large-scale RCTs with BDD and other disorders with elevated STB risk.

Active, acute suicidal ideation was assessed via QIDS-SR item 12. A score>0 triggered a “pop up” message presented to the patient within the mobile app reminding them that if they were experiencing suicidal thoughts they should seek professional help without delay or if they felt unsafe to call 911 or go to their nearest emergency room. Links to 911 and the national suicide hotline were provided within this pop-up notification. Here, we note that a recently published expert consensus statement [17] on managing suicide risk in digital studies indicates that most suicide experts polled agreed it was appropriate to present pop-ups containing resources like 911 and the national suicide hotline to participants with elevated suicide risk, where risk is typically determined by the presence of suicidal thoughts or current intent>0. However, most experts also agreed that pop-ups should not expressly suggest that all participants with any or all suicidal thoughts should go to their nearest emergency room, as this would be unwarranted for many and potentially iatrogenic for some. Instead, the decision to initiate a higher level of care or active rescue should be determined via clinical outreach. Therefore, we would advocate future studies follow this updated guidance. A score>1 triggered an alert to the clinician and coach. A study clinician followed up with a phone evaluation within 24 hours of an alert. Risk was assessed via clinical interview, referencing the participant’s recently reported scores and asking follow-up questions derived from validated measures (eg, C-SSRS and Beck Depression Inventory) related to the frequency and intensity of suicidal thoughts, presence and specificity of suicide planning and preparation, level of current suicide intent and access to means, previous STB and current impairment and distress. Clinicians completed a safety plan with participants when warranted by increased death or suicide-related thoughts or risk of suicidal behavior. Participants were to be referred to a higher level of care, including going to an emergency room, if clinically indicated by this risk assessment.

### Data Analyses

To examine the differences between participants included versus excluded from the trial, we computed a *t* test with unequal variance to compare mean depression severity and a chi-square test to compare prevalence of death- and suicide-related thoughts collected at screening. To characterize participants’ lifetime STB, we computed descriptive statistics (eg, mean and percentage) on C-SSRS variables collected at baseline. To calculate the incidence of STB during the trial, we took several steps. First, we aggregated all weekly, mid, and endpoint QIDS-SR item #12 responses. There were 1440 possible observations, of which 397 (27.5%) were missing. Second, we created three dichotomous variables: (1) to capture any “life not worth living, death- or suicide-related thought,” we recoded item responses as 0 versus ≥1 (2) to capture “several death- or suicide-related thoughts in any week,” we recoded responses as ≤1 versus ≥2 and (3) to capture increased severity of death- or suicide-related thoughts, we evaluated whether any subsequent QIDS-SR item #12 response was>baseline response (increased severity) versus whether any subsequent response was ≤ the baseline response (no increase in severity). Third, we calculated descriptive statistics (eg, count and percentage) for each variable. To predict STB incidence, we ran a series of bi- and multivariate logistic regressions to predict dichotomous outcomes. Bivariate models predicted STB with either (1) lifetime suicidal ideation intensity or (2) lifetime suicide attempt, both collected via the C-SSRS at baseline. Multivariate models included the following baseline variables: birth sex (female: n=67, 83.8%), age (mean 27, SD 9.64; C-SSRS lifetime suicidal ideation severity (mean 1.83, SD 1.86, median 1, IQR 3.25), lifetime suicide attempt (n=8, 10%), QIDS-SR depression severity total score, excluding the death or suicide related thoughts item #12 (mean 10.94, SD 3.90), BDD symptom severity (BDD Y-BOCS, mean 30.35, SD 4.4), and sexual orientation. Sexual orientation was a categorical variable with three levels: “Straight/Heterosexual” (n=49, 61.3%), “Lesbian, Gay/Homosexual, or Bisexual” (n=20, 25%), and “Other,” (n=11, 13.8%), which includes participants endorsing sexual orientation options, “Something else,” “don’t know,” or “choose not to disclose.” We included these covariates because they are known risk factors of STB [8]. We included both bi- and multivariate models to evaluate whether single variables (eg, suicide attempt) or a collection of variables would be helpful in predicting which trial participants would report STB during the treatment phase. All analyses were carried out using R Statistical Software (version 4.4.2; R Core Team 2021) [32].

### Ethical Considerations

Participants provided electronic, written informed consent before the initiation of any study procedures. The study protocol was reviewed and approved by the Mass General Brigham institutional review board (approval number 2017P000293).

## Results

### Reasons for Exclusion

A total of 107 people were screened for the trial. Of those, 27 (25.2%) were not included for the following reasons: 14 (13.1% of those consented) for a nonprimary BDD diagnosis, 4 (3.7%) for endorsing acute suicidal ideation in the past month (ie, active suicidal ideation as indicated by clinical judgement or a score of 2 or greater on the suicidal ideation subscale of the C-SSRS), 4 (3.7%) for not meeting criteria for BDD, 4 (3.7%) for having received greater than 3 sessions of CBT, 3 (2.8%) for bipolar symptoms, 3 (2.8%) for declining to participate (eg, due to life stress and learning more about the treatment offered), and 1 (0.9%) for acute depression, as indicated by clinical judgement or a QIDS-SR total score of 21 or greater.

### Differences Between Participants Excluded Versus Included

Participants included in the study did not significantly differ from those excluded in past-week depression severity (QIDS-SR, total mean 11.28, SD 4.14 vs mean 12.44, SD 3.90; *t*=1.33; Cohen *d*=–0.29, 95% CI –0.73 to 0.16; *P*=.19), nor in prevalence of past-week death- or suicide-related thoughts (ie, >0 on QIDS-SR item 12; n=26, 32.5% vs n=12, 44.4%; *χ*²_X_=1.25; *P*=.26). Demographic and other data were not collected for people deemed ineligible during screening to reduce burden.

### Suicide Risk Alerts and Outcomes

During the treatment phase—that is, the first 3 months for participants randomized to immediately receive the app and the first 6 months for those randomized to the waitlist—12 risk alerts were triggered by 11 different participants. All risk alerts were generated from responses to weekly in-app surveys. Around 10 alerts were related to elevated death- or suicide-related thoughts and 2 alerts were related to increased clinical severity (1 for depressive symptoms and 1 for BDD symptoms). Per protocol, study clinicians followed up with participants via telephone. In all but one instance, participants were reached within hours of triggering the alert. With those reached, a study clinician completed an in-depth risk assessment and created or reviewed a safety plan. All participants were deemed to be at low risk for attempting suicide in the near future and as such did not require a higher level of care or withdrawal from the study. The participant who was not reached after multiple attempts (via telephone and email) was monitored for responsiveness to in-app notifications and engagement (eg, completion of CBT exercises). This participant continued to engage with treatment and did not meet criteria for the study team to call their emergency contact. However, due to nonresponsiveness and increased clinical severity, this participant was withdrawn from the study and provided symptom-specific resources and clinical referrals via email.

### Trial Participants’ Lifetime and Past-Month Prevalence of STB as Assessed by C-SSRS

#### Lifetime

At baseline, most trial participants (65.0%, n=52) reported experiencing a wish to die at some point in their lives (C-SSRS, Q1). Just under half (41.3%, n=33) reported nonspecific active suicidal thoughts (Q2), 37.5% (n=30) reported active thoughts without a plan or intent (Q3), 20% (n=16) reported active suicidal thoughts with some intent but without a specific plan (Q4), and 13.5% (n=11) reported active suicidal thoughts with some intent and a specific plan (Q5). About 10% (n=8) of participants reported having attempted suicide, 16% (n=13) reported engaging in some form of suicidal behavior (ie, suicide preparation or aborted, interrupted, or actual suicide attempt), and 30% (n=24) reported past nonsuicidal self-injury. These results are in line with previous literature indicating that STB is relatively common among people with BDD [21].

#### Past Month

A total of 25% (n=20) of participants reported a wish to die (Q1) in the month before starting the trial. No participants reported experiencing more severe suicidal thoughts, as this was an exclusion criterion.

#### Incidence of STB During the Trial

Table 1 displays the incidence of STB during the trial. Notably, no participants reported experiencing daily suicide- or death-related thoughts or making a specific suicide plan. However, 20 (25%) participants reported that their death- or suicide-related thoughts were worse during the trial relative to the week before baseline, and only one participant reported that these thoughts were worse or more severe during the trial relative to their lifetime.

**Table 1.** Incidence, new onset, and increased severity of participants’ death and suicide-related thoughts or behaviors during the 3- to 6-month treatment phase of a smartphone-delivered treatment trial for body dysmorphic disorder (BDD, N=80).

Variable	Participants, n (%)
Incidence	34 (42.5)
Life feels empty or I wonder if worth living	24 (30.0)
Several suicide- or death-related thoughts this week	10 (12.5)
New onset	3 (3.8)
Life feels empty or I wonder if worth living	2 (2.5)
Several suicide- or death-related thoughts this week	1 (1.3)
Increased severity	
Relative to the week before baseline^a^	20 (25.0)
Relative to lifetime^b^	1 (1.3)
Suicide attempt	0 (0)
Withdrawn for worsening symptoms or suicidal thoughts	1 (1.3)

a Greatest severity in the week before baseline as assessed by QIDS-SR (Quick Inventory of Depressive Symptomatology-Self-Report).

b Greatest lifetime severity as assessed by C-SSRS (Columbia-Suicide Severity Rating Scale) at baseline.

#### Predicting STB

Table 2 presents the results of logistic regressions using baseline variables to predict the incidence of death- and suicide-related thoughts during the 12-week trial. In the leftmost column, bivariate models revealed that higher severity of suicidal thoughts and, separately, reporting a previous suicide attempt at baseline were significant predictors of reporting any death- or suicide related thought during the trial. However, after adjusting for covariates known to be associated with STB (eg, age, birth sex, and sexual orientation), only baseline suicide-thought severity remained a significant predictor of death- or suicide thought incidence. Similarly, bivariate models presented in the middle column revealed that both higher baseline suicide-thought severity and lifetime suicide attempt significantly predicted experiencing several death- or suicide-related thoughts for several minutes in any given week. However, these relationships did not remain significant after adjusting for covariates related to STB risk. Bivariate models presented in the rightmost column revealed that only baseline suicide-thought severity predicted which participants would report an increase in the severity of death- or suicide-related thoughts during the trial, relative to the week before baseline. No variables were significant predictors of this outcome after adjustment for covariates.

**Table 2.** Incidence of death- and suicide-related thought outcomes during the three-month treatment phase of a smartphone-delivered treatment trial for body dysmorphic disorder (BDD, N=80).

Model and predictors	Dichotomous outcomes occurring during the 12 wk trial assessed by weekly QIDS-SR16 item 12^a^																				
	Any life feels empty, death- or suicide-related thoughts^b^ (n=34)							Several death- or suicide-related thoughts in any week^c^ (n=10)							Increased severity of death- or suicide-related thoughts^d^ (n=20)						
	OR^e^	95% CI	*P* value	PPV^f^	NPV^g^	AUC^h^	*R* ^2^	OR	95% CI	*P* value	PPV	NPV	AUC	*R* ^2^	OR	95% CI	*P* value	PPV	NPV	AUC	*R* ^2^
Lifetime suicide-thought severity^i^	1.61	1.24‐2.14	.001	.70	.63	67	0.160	1.76	1.21‐2.77	.006	—^j^	—	—	0.117	1.31	1.00‐1.74	.050	—	—	—	0.047
Lifetime suicide attempt (yes)	11.67	1.93‐224.35	.025	.63	.88	.75	0.092	11.00	2.14‐59.14	.004	.92	.50	.71	0.143	3.50	0.75‐16.37	.100	.78	.50	.64	0.037
Adjustment for covariates	—	—	—	.70	.69	.69	0.236	—	—	—	.91	.75	.83	0.244	—	—	—	.76	.50	.63	0.073
Birth sex (M)	1.74	0.37‐7.97	.470	—	—	—	—	0.00	0.00‐100+	.994	—	—	—	—	1.17	0.21‐5.24	.845	—	—	—	—
Age	0.97	0.91‐1.02	.279	—	—	—	—	0.97	0.84‐1.08	.660	—	—	—	—	0.98	0.91‐1.05	.605	—	—	—	—
Sexual orientation: LGB^k^	1.25	0.35‐4.48	.724	—	—	—	—	0.82	0.11‐5.22	.834	—	—	—	—	1.01	0.27‐3.60	.986	—	—	—	—
Sexual orientation: Other^l^	0.48	0.09‐2.22	.366	—	—	—	—	0.44	0.02‐4.40	.531	—	—	—	—	0.57	0.07‐2.89	.528	—	—	—	—
Lifetime suicide-thought severity	1.45	1.04‐2.07	.034	—	—	—	—	1.55	0.90‐2.93	.132	—	—	—	—	1.34	0.92‐1.99	.135	—	—	—	—
Lifetime suicide attempt (yes)	3.40	0.35‐80.14	.339	—	—	—	—	3.19	0.30‐37.54	.335	—	—	—	—	1.23	0.17‐8.98	.837	—	—	—	—
Baseline depression severity^m^	1.12	0.97‐1.32	.148	—	—	—	—	1.11	0.86‐1.47	.450	—	—	—	—	0.99	0.85‐1.16	.934	—	—	—	—
Baseline BDD severity	1.06	0.93‐1.21	.408	—	—	—	—	0.86	0.68‐1.09	.221	—	—	—	—	0.95	0.81‐1.09	.444	—	—	—	—

a QIDS-SR16: Quick Inventory of Depressive Symptomatology-Self Report (16-item); item 12 concerns “Thoughts of death or suicide.”

b Negative response to QIDS-SR item 12 option 0: “I [did] not think of suicide or death.”

c Affirmative response to QIDS-SR item 12 option 2: “I think of suicide or death several times a week for several minutes.”

d Severity increasing relative to baseline death- and suicide-related thoughts assessed via QIDS-SR item 12.

e OR: odds ratio.

f PPV: positive predictive value.

g NPV: negative predictive value.

h AUC: area under the receiver operator characteristic curve.

i Lifetime suicide thought severity assessed via the C-SSRS at baseline.

j Not available.

k LGB, Lesbian, Gay/Homosexual, Bisexual (*n*=20).

l Other, includes participants (n=11) endorsing sexual orientation options “Something else,” “don’t know,” or “choose not to disclose.”

m Baseline depression severity assessed via the QIDS-SR16 excluding item 12, as this item was used to create death-/suicide-related thought outcome variables.

## Discussion

### Principal Findings

The purpose of this study was to examine the rates of STBs in participants of a recent RCT of smartphone-delivered CBT for BDD. There were four main findings. First, those included versus excluded did not significantly differ in past-week depression severity or the prevalence of past-week death- or suicide-related thoughts. Second, consistent with the results of previous research (eg, 21), a lifetime [27] history of relatively severe STB was prevalent among this sample of participants with primary BDD, with many reporting previous active suicidal thoughts (40%) and a previous suicide attempt (10%). Third, over the course of the three-month treatment phase, nearly half of the participants reported experiencing a death- or suicide-related thought, but no participants experienced acute and frequent suicidal thoughts, made a suicide plan, or attempted suicide. Fourth, previous suicide attempt and the severity of suicidal thoughts were significant predictors of who would experience death- or suicide-related thoughts during the trial. However, only baseline suicide thought severity remained a significant predictor after adjusting for known STB risk factors (eg, demographics and sexual orientation). Notably, even the best models had only relatively modest predictive accuracy (eg, AUCs ranging from 0.63 to 0.83), highlighting the difficulty of short-term prediction of STB [9].

The main implication of these findings is that with carefully selected eligibility criteria and robust yet relatively low burden and ongoing monitoring procedures, it is possible to conduct a safe, light-touch DMHI, even when including some participants (eg, those with past STB) with moderately elevated risk profiles. Despite excluding participants with “active” STBs occurring in the past month, a meaningful proportion of participants with a psychiatric disorder such as BDD may still experience death- or suicide-related thoughts during a several months long treatment trial, particularly those with previous acute STB, such as suicide attempt or active suicidal ideation. Importantly, despite these fluctuations in suicidal thoughts, no participants reported attempting suicide. Together, this highlights the importance of assessing STB on an ongoing (eg, weekly) fashion that imposes low burden on participants but also provides actionable data to the research team. Questions and answer options that are concise and easily understood are always preferrable, especially so in DMHIs, because participant adherence and engagement tends to decline precipitously as treatments progress [33]. Items with high face and construct validity that researchers can use to quickly inform decisions about when to follow-up with participants are essential in DMHIs, given these interventions are remotely administered and have historically suffer from low engagement, limiting opportunities for observing and assessing risk relative to more traditional, face-to-face treatments.

The findings from this study must be interpreted in the context of several limitations. First, nearly a third of participants’ weekly death- and suicide-related thought responses were missing. Although this amount of missing data is on the low end compared with other DMHIs [33], any missing data limits our ability to estimate the true incidence of death- and suicide-related thoughts. Future studies could attempt to address this by making some proportion of weekly surveys mandatory or by incentivizing their completion. However, the former runs the risk of increasing participant burden and, therefore, decreasing adherence, whereas the latter may not be financially feasible in real-world implementations, limiting the generalizability of findings. Second, the item we chose to assess weekly death- and suicide-related thoughts included answer options that vary in the constructs they assess (eg, three options specifically mention death- and suicide-related thoughts, one does not, two options assess frequency, and two do not), limiting precision. We chose this item to maximize the trade-off between high face validity and low participant burden, while matching our a priori risk-mitigation decision rules for when to actively check in with participants to assess current risk. Future studies could attempt to pilot and validate additional items with more favorable precision. Additionally, assessing the presence of death-or suicide-related thoughts with just one item can lead to misclassification of suicide risk [34,35]. Future studies could assess risk with additional items but must be mindful of balancing the benefits of increased precision with the costs of increased burden and the potential for resulting lower adherence, which can also lead to misclassification. Third, we excluded participants (n=4) with “active” suicidal thoughts or intent in the past month, given the light-touch nature of the treatment. In doing so, it is possible that some participants were needlessly excluded, thus limiting the generalizability of the treatment and the risk assessment and mitigation procedures reported here. Using this potentially overly restrictive criterion also runs the risk of perpetuating treatment barriers. If future studies implement less restrictive STB exclusion criteria, they may find it helpful to make risk assessment and mitigation procedures more robust (eg, increase assessment frequency and alter “risk-alert” thresholds to be more sensitive).

### Conclusion

Our findings indicate that it is feasible to conduct an RCT of a DMHI among a sample of clinically acute participants with relatively high rates of lifetime STB. There are inherent risks in conducting treatment trials and trade-offs when establishing thresholds for eligibility, particularly for highly prevalent and stigmatized symptoms such as STB. Trials with very strict eligibility criteria may effectively limit the risk of STB, but findings will likely lack generalizability and potentially perpetuate treatment barriers for patients in high need. Trials with overly lenient eligibility criteria may fail to mitigate the risk of serious adverse events, jeopardizing participant safety, trial feasibility, and the implementation of an otherwise promising treatment. Researchers can include participants with moderately elevated risk profiles by carefully selecting eligibility criteria based on rational and empirical grounds, carefully screening participants with trained study staff, using frequent, ongoing, low burden, and valid monitoring procedures, implementing risk mitigation protocols that are tailored to the presenting problem, ensuring staff are adequately supervised and trained to handle anticipated STB and adverse events as well as active risk mitigation efforts, and that patients are aware of these procedures and understand why they are answering such questions. More research should examine the safety procedures implemented in RCTs and report more fully on adverse events and other key outcomes such as STB. Doing so will help ensure that, as a field, we are following adequate and generalizable risk mitigation practices, while not needlessly excluding participants who could benefit from treatment.

## Abbreviations

BDD: body dysmorphic disorder
C-SSRS: Columbia-Suicide Severity Rating Scale
CBT: cognitive behavioral therapy
CGI-BDD: Clinical Global Impressions scale adapted for assessing BDD
DMHI: digital mental health intervention
*DSM-5*: *Diagnostic and Statistical Manual of Mental Disorders* (Fifth Edition)
MINI: Mini International Neuropsychiatric Interview
PHQ-2: Patient Health Questionnaire-2
PI: principal investigator
QIDS-SR: Quick Inventory of Depressive Symptomatology-Self Report
RCT: randomized control trial
STB: suicidal thoughts and behavior
YBOCS: Yale-Brown Obsessive Compulsive Scale
